# Supplementary figures and images for: Small hand-designed convolutional neural networks outperform transfer learning in automated cell shape detection in confluent tissues
Source: PLoS One. 2023 Feb 16;18(2):e0281931. doi: 10.1371/journal.pone.0281931 (PMC9934364; doi:10.1371/journal.pone.0281931)

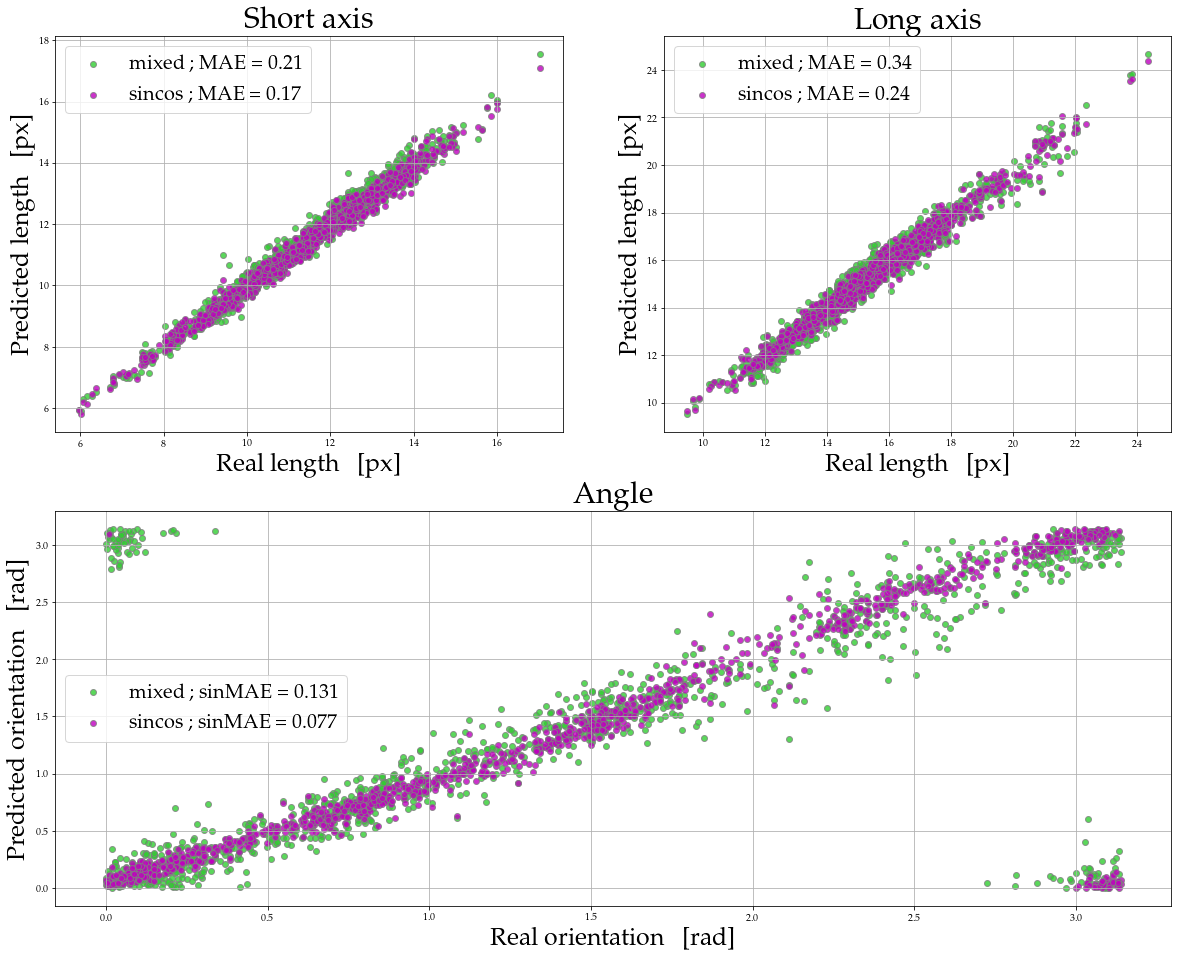

Supplement: S1 Fig — Comparison of performance of a simple 2 convolutional layer CNN trained either with the raw orientation and a circular MSE (mixed) or by encoding orientation as sine and cosine (sincos). In each case, the corresponding MAEs are shown in inserts. (TIF) [file pone.0281931.s001.tif]

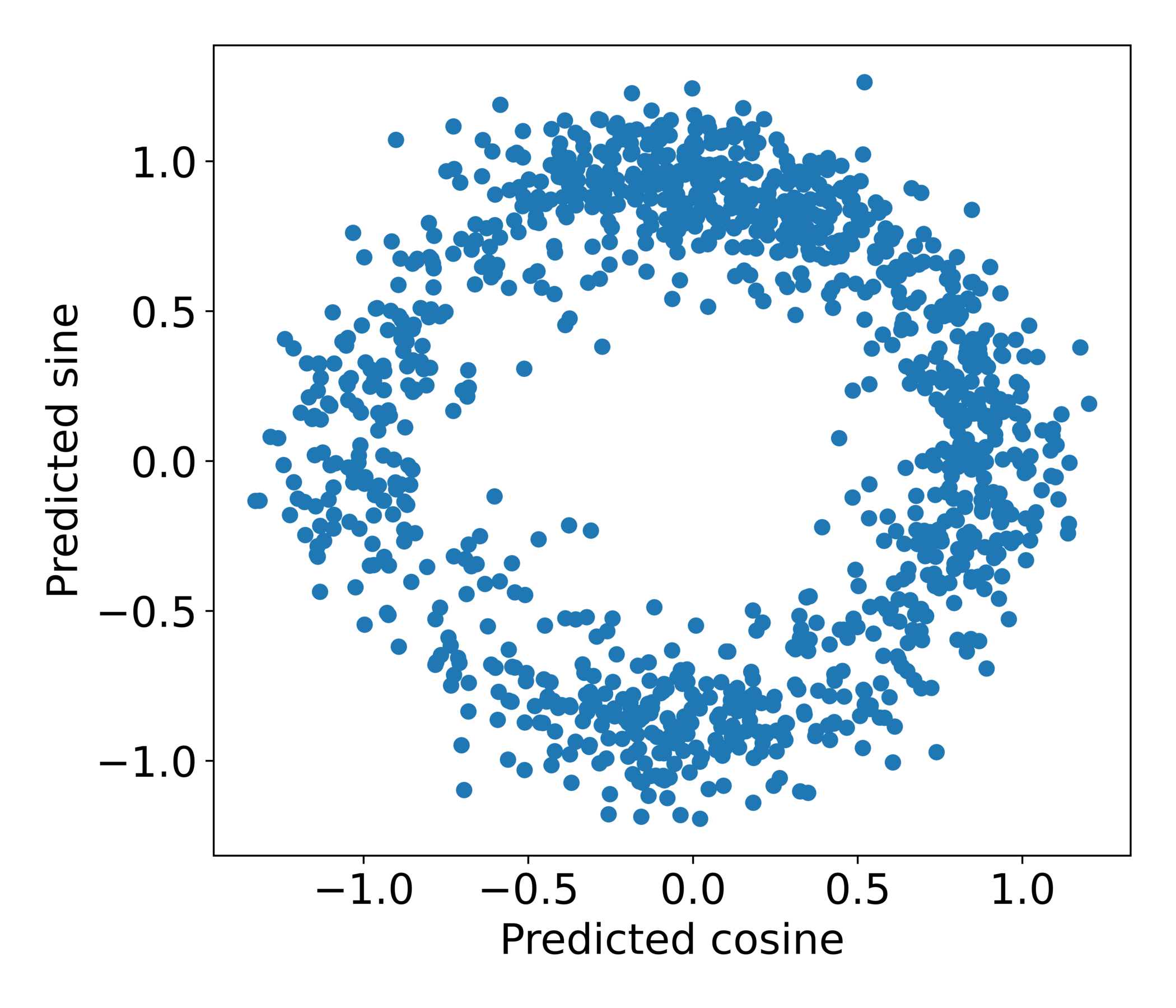

Supplement: S2 Fig — Correlation between predicted sines and cosines from a 2-conv layer CNN on the test set. (TIF) [file pone.0281931.s002.tif]

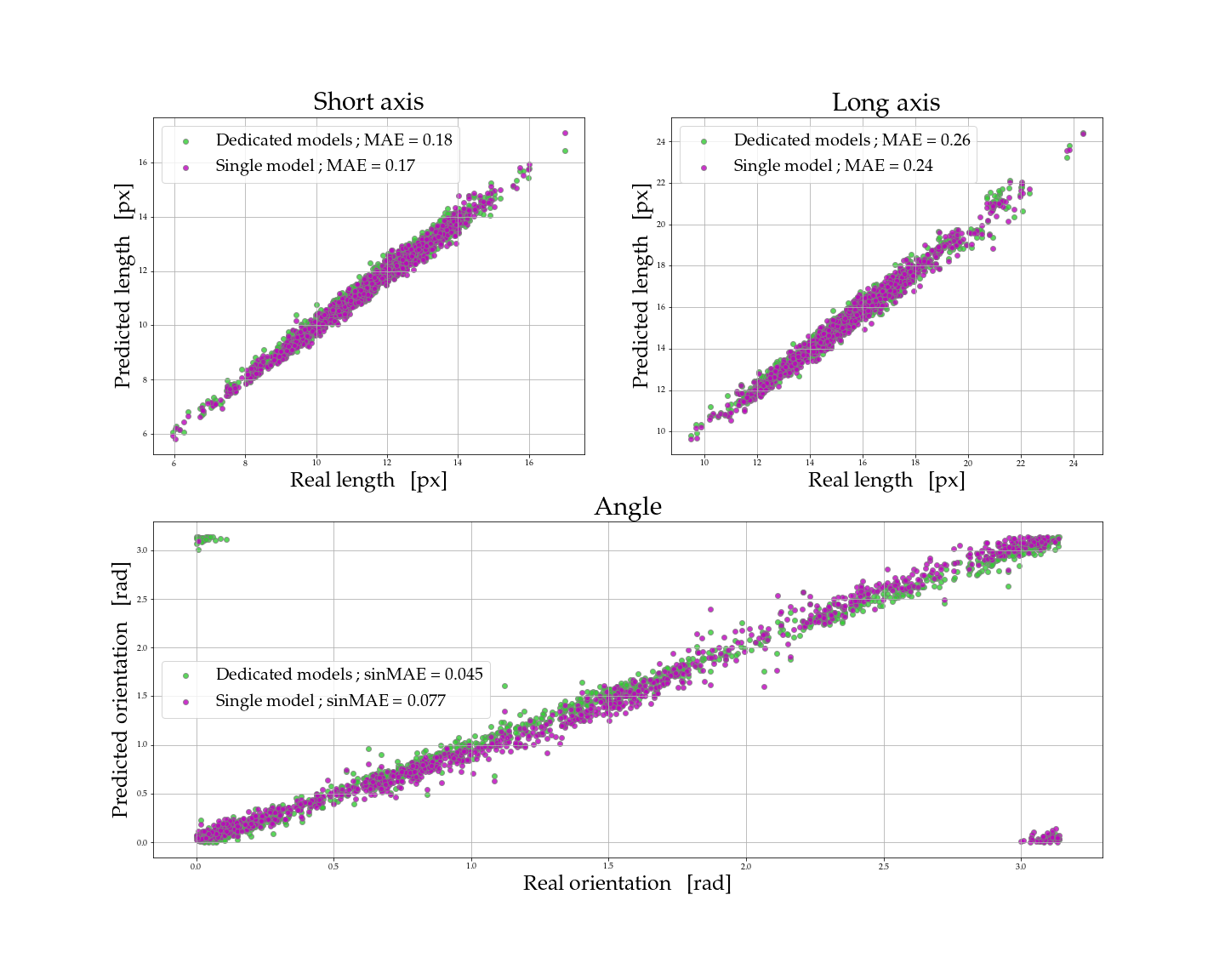

Supplement: S3 Fig — Comparison of performance of the performance of simple 2-conv CNNs. The dedicated models are two different CNNs, one trained to predict lengths only and the other orientation only. The single model predicts all three quantities at once. In each case, the corresponding MAEs are shown in inserts. (TIF) [file pone.0281931.s003.tif]

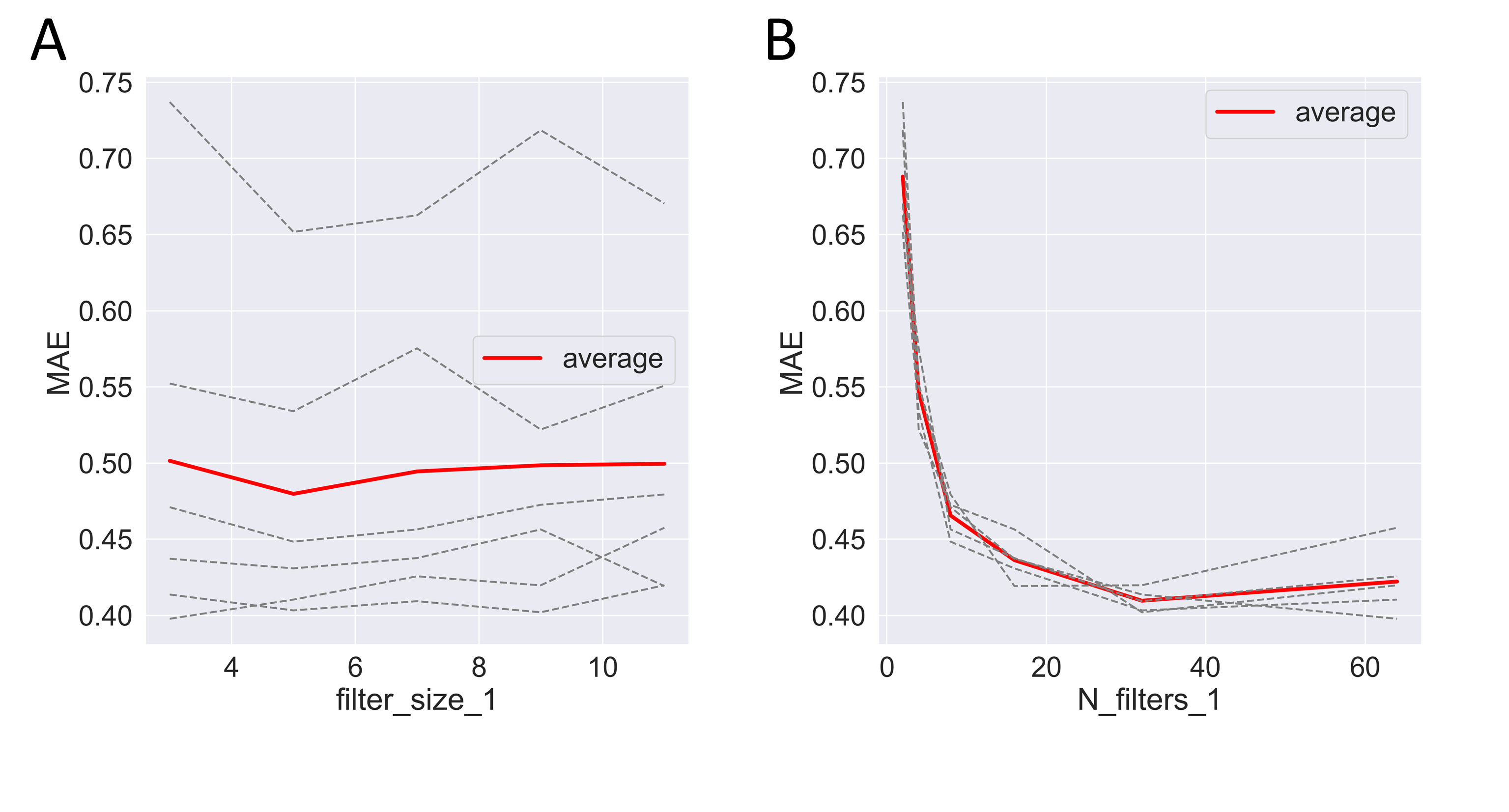

Supplement: S4 Fig — In A, each dashed black line corresponds to one value of the number of filters and the red line is the average of all black lines. In B, each dashed black line correspond to one value of filter size and the red line is the average of all black lines. (TIF) [file pone.0281931.s004.tif]

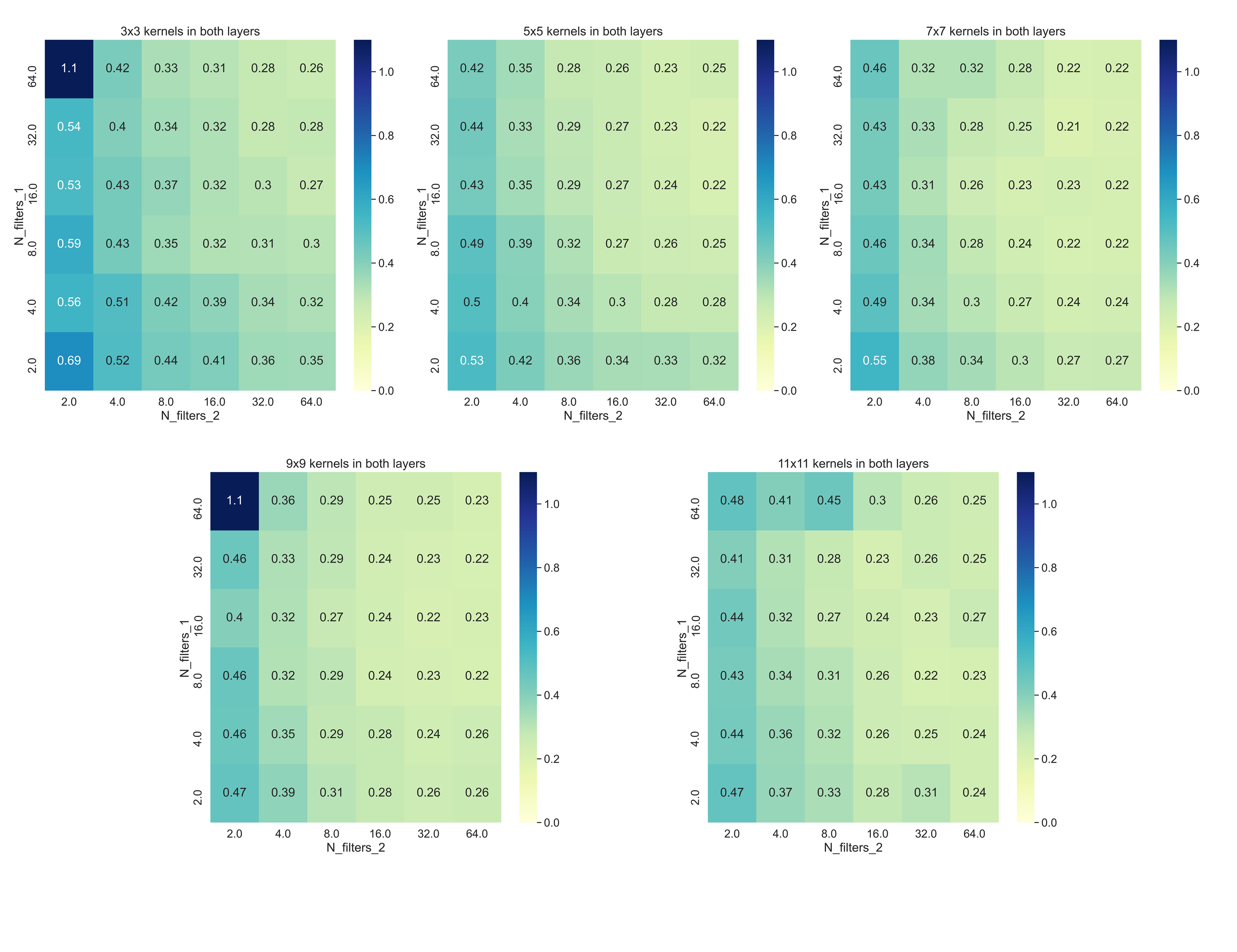

Supplement: S5 Fig — Each graph shows models where both layers have the same filter sizes (from 3 to 11). Each value of the MAE averaged over 5-fold is shown both in colors and numbers and corresponds here to a single network architecture, or a single model. (TIF) [file pone.0281931.s005.tif]

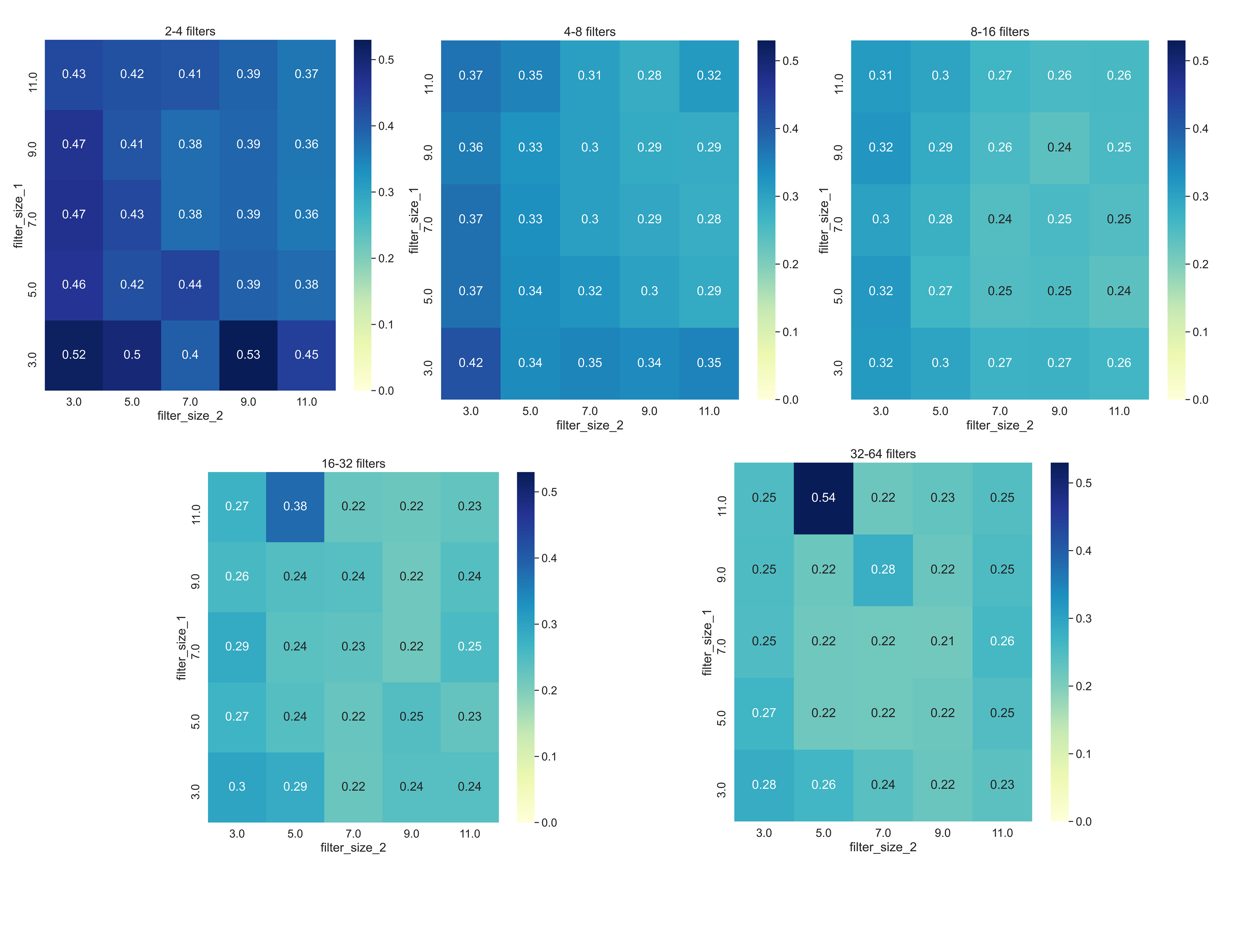

Supplement: S6 Fig — Same analysis as Fig SI5 but for the sizes of the filters. Each model in a graph respects the rule that the number of filters is multiplied by 2 between the two layers and these values span from 2–4 to 32–64. (TIF) [file pone.0281931.s006.tif]
